# Promoting Sustainable Work Hours Through Participatory Scheduling: A Cross‐Sectional Study Employing Objective Working Time Data

**DOI:** 10.1155/jonm/4164713

**Published:** 2026-08-17

**Authors:** Philip Tucker, Majken Epstein, Anna Dahlgren

**Affiliations:** ^1^ Department of Psychology, Stockholm University, Stockholm, Sweden, su.se; ^2^ School of Psychology, Swansea University, Swansea, UK, swansea.ac.uk; ^3^ Department of Clinical Neuroscience, Division of Psychology, Karolinska Institute, Solna, Sweden, ki.se

## Abstract

**Background:**

Participatory working time scheduling (PWTS) is a form of self‐scheduling involving collaboration between employees and employers. PWTS is common in Swedish health care, but its characteristics can vary substantially between workplaces. This study investigated whether the characteristics of PWTS were associated with (1) the ergonomic characteristics of the schedules that emerge from this process and (2) employee satisfaction with those schedules—two indices reflecting the sustainability of work hours.

**Methods:**

Two hundred and forty‐eight nurses, assistant nurses and care assistants (response rate 29%) from two Swedish hospitals completed a survey that asked about the respondents’ experience of how the scheduling process is managed and implemented, and their own priorities when planning their schedules. Participants’ realised work hours were derived from the hospitals’ administrative systems. Factor analysis was used to identify composite factors of PWTS characteristics. Logistic regressions then examined associations between PWTS characteristics and (1) the prevalence of ergonomically unsound work schedule characteristics and (2) indices of employee satisfaction.

**Results:**

Six PWTS characteristics were identified: scheduling support and personal influence (SSPI), prioritising personal motives (PPM), usability of scheduling tools (UST), scheduling as a collective process (SCP), preference for compressing hours (PCH) and bad schedule warnings (BSW). SCP and PCH predicted the frequency of long (> 40 hours) working weeks. BSW predicted the frequency of long shifts (≥ 12 h). SSPI predicted satisfaction with work hours, satisfaction with influence over work hours and frequency of thoughts about leaving the workplace.

**Conclusions:**

The findings indicated that, for PWTS to be perceived positively, there should be open communication between employees and managers/staffing assistants, fostering individual relationships with employees and promoting employee‐voice in the scheduling process such that nurses feel able to influence their work hours. However, we found only limited evidence that PWTS characteristics affected the ergonomic characteristics of the schedules that emerged from this process.

**Implications for Nursing Management:**

Priority should be given to allowing staff influence over their work hours while simultaneously providing those staff with support in the scheduling process. Managers need to be active in their leadership of work hours, so as to foster that communication and employee‐voice. Managers should identify staff who choose to work longer work weeks and alert them to the potential risk to their own wellbeing and to patient safety. An important tool in this regard is scheduling software that warns of ergonomically unsound schedules.

## 1. Introduction

Healthcare today faces major challenges in recruiting and retaining staff [1]. Providing attractive and sustainable working hours is an important tool in meeting this challenge [2]. Giving shiftworking nurses influence over their working hours, through self‐scheduling, is an important means of achieving this. Self‐scheduling has been defined as a method that allows employees a certain amount of flexibility in choosing their shift patterns, which management can augment in terms of coping with operational requirements [3]. Self‐scheduling aims to enhance employees’ work time control (WTC), thereby offering the potential to mitigate the impact of demanding work schedules. Enhanced WTC can facilitate a better work–life balance, allowing the employee to align their work commitments with their private life [4]. Self‐scheduling can also improve employees’ experience at work, e.g., positive effects on job demands and the social environment at work [5]. Such enhancements may underlie observed associations between self‐scheduling and improved employee satisfaction, health and well‐being. Self‐scheduling also offers potential benefits at the organisational level, including improved scheduling efficiency (e.g., through reductions in shift change requests, roster adjustments and shift swaps), reduced sickness absence, and improved staff retention and turnover [6].

Despite the potential benefits of self‐scheduling, it also carries potential risks to employees and organisations. Workers may use the autonomy to choose ‘unhealthy’ or more fatiguing working hours, for example, by prioritising social activities or financial gain over recovery, sleep and health [7]. Some studies have found that implementing self‐scheduling led to increased long work shifts [3, 8]. A range of shift schedule characteristics are associated with impairments of sleep and subsequently heightened fatigue, including high frequency of short intershift intervals (< 11 h, so‐called ‘quick returns’), frequent night shifts, working many consecutive shifts (especially night shifts; e.g., ≥ 4), long shifts (e.g., ≥ 12 h), long weekly work hours and having only a single day off after nightwork [9]. Moreover, long‐term exposure to such schedule characteristics, and the associated chronic disruption of sleep and circadian rhythms, has been linked to increased risk for the development of certain chronic diseases, e.g., Type 2 diabetes, coronary heart disease and cancer [10, 11].

From an organisational‐risk perspective, self‐scheduling may result in employees prioritising longer continuous free time at the cost of operational functionality of the work unit [12]. Individualistic self‐scheduling may diminish the cohesion of well‐established work teams. It may lead to competition and conflicts among colleagues for the more desirable shifts, with risk of less assertive employees’ wishes being overlooked [13]. The relationship between the manager and staff may be negatively affected, e.g., when scheduling decisions are perceived as unfair [14]. Self‐scheduling can be perceived by staff as being less fair than traditional scheduling (e.g., manager‐led scheduling [15]) and has been shown to be negatively associated with procedural and organisational justice [14]. This, in turn, could negatively impact staff retention, with unequal shift assignment and perceived lack of procedural justice having been linked to greater turnover intention [16].

These potential downsides of self‐scheduling highlight the increased responsibility that it places on the individual employee in the scheduling process. Employees who share this responsibility need education to promote their understanding of the scheduling problem, as well as good communication skills and assertiveness to negotiate with colleagues and managers [7, 15, 17].

While the majority of studies have reported positive effects of self‐scheduling, for both employees and for the organisation, there are conflicting findings [6]. Such inconsistencies may reflect the heterogeneous nature of the self‐scheduling concept. Traditional individualistic forms of self‐scheduling allow the worker a degree of flexibility in choosing their own shift patterns, subject to adjustment by managers to meet operational requirements. In contrast, participatory working time scheduling (PWTS) is a collaborative approach to schedule planning involving cooperation between management and individual employees, often taking place in several steps and in cycles of negotiation and adjustments, mediated by scheduling software. In this approach, nurses’ work hours are planned to meet both individual requests for working hours and the employers’ needs in terms of skills and staffing, taking into consideration working time legislation, operation of the ward, fairness and equality. PWTS is thus both an opportunity and an obligation for the employee to participate in their own scheduling and their coworkers’ scheduling [3].

PWTS is common in Swedish and Finnish healthcare. In Swedish healthcare settings, the process typically starts with a planning period (1–10 weeks), when employees propose which shifts they wish to work during the next period, usually using a scheduling software application. There are often rules and restrictions about which shifts are worked (e.g., minimum number of weekend shifts). The employee may veto specific shifts which she/he does not wish to work (the maximum number of vetoes varies). Then, an adjustment period (1–3 weeks) follows when changes are made to fulfil staffing needs on each shift, usually carried out by the employees themselves and/or staffing assistants. After this, staffing assistants sometimes do further adjustments (1–3 weeks), before the manager finally approves the schedule [18].

In an earlier qualitative study [18], we examined how healthcare managers and staffing assistants work to achieve sustainable work hours within the context of PWTS. We defined sustainable working hours as working hours that promote both short‐ and long‐term employee health, sleep and recovery, as well as patient safety. We found that the scheduling process benefited when there was a shared responsibility framework (i.e., active engagement by all persons involved in the scheduling process) and that problems emerged when employees did not engage sufficiently in the scheduling process. Having an open dialogue regarding work hours was considered by interviewees to be important for facilitating mutual problem solving and discussions about the importance of sustainable scheduling. Fairness in the distribution of unpopular shifts was seen to be a key issue. In particular, distribution of evening shifts and working on public holidays were identified as contentious issues, with dissatisfaction being managed by explaining and giving a rationale for shift changes. The importance of providing employees with education, ongoing support and clear rules in relation to scheduling was emphasised. While the importance of giving employee’s freedom in the scheduling process was acknowledged by interviewees, it was noted that some employees would self‐schedule unsustainable work hours, such as compressing shifts to get longer continuous periods of time off. Managers who saw scheduling as an important part of their leadership role felt that having insight into their employee’s schedule and their personal circumstances made the scheduling process smoother. Lastly, the design and performance of the technological systems used in the scheduling process were identified as potentially crucial enablers or barriers for achieving sustainable work hours.

In conducting the above study, we observed that PWTS characteristics could vary substantially between workplaces (e.g., division of responsibilities, scheduling guidelines, technical support, use of vetoes, leadership and employee engagement). We concluded that the conditions under which PWTS is implemented (i.e., the characteristics of the PWTS process itself and the characteristics of the work environment in which it is implemented) may affect the chances of achieving attractive and sustainable working hours. Nevertheless, it remained unclear precisely how such differences in conditions might affect the long‐term sustainability of working schedules. To address this, the current study investigates whether characteristics of PWTS implementation are associated with two indices reflecting the sustainability of work hours: (i) the ergonomic characteristics of the schedules that emerge from this process and (ii) employees’ satisfaction with their work hours. In addition to these primary analyses, secondary analyses are conducted to investigate whether characteristics of PWTS implementation are associated with job satisfaction, satisfaction with influence over work hours and having thoughts about leaving the current workplace.

## 2. Materials and Methods

### 2.1. Design

A cross‐sectional questionnaire survey formed the basis of this study. The initial stage of the analysis sought to identify PWTS characteristics through exploratory factor analysis (EFA). The next stage tested the primary hypotheses concerning associations between the PWTS characteristics identified in the first stage as predictors and predefined measures of work schedule characteristics and satisfaction with work hours as outcomes. This stage also included conduct of the secondary (regression) analyses outlined above. The study reporting adhered to the STROBE checklist for cross‐sectional studies (see supporting file 1).

### 2.2. Participants and Data Collection

Participants were recruited at 12 units within two hospitals in different Swedish regions. The inclusion criteria were to be working as a nurse, nursing assistant or care assistant in a unit that utilised PWTS. Email addresses of potential participants were obtained through the human resources department. An email was sent containing information about the study and giving recipients the possibility to provide informed consent digitally, via the tool Artologik. After informed consent was given, participants were given access to the digital questionnaire. Between 3 and 10 reminders were sent out after the initial invitation. The questionnaire was completed by 248 (response rate 29%) participants between November 2022 and September 2023. Objective working time data were obtained from the hospitals’ administrative systems for 238 participants, with data for 10 of the 248 survey participants having been lost due to a technical malfunction.

#### 2.2.1. Questionnaire Survey

The questionnaire included three sets of items that were the basis of the current analyses, along with the objective working time data (see below). The first set of items sought to characterise the respondents’ experience of the PWTS process in their workplace. The items were based on interviews conducted with staffing assistants and first‐line managers in Swedish healthcare settings [18], as described in the introduction. The items focussed on how the scheduling process was managed and implemented, and on the importance that respondents placed on personal priorities when planning their schedules. The second set of items assessed respondents’ perceptions of their work environment, namely the perceived sufficiency of staffing levels, satisfaction at work and job tempo. The third set of items obtained demographic information.

Outcomes and operationalisations of variables were preregistered (https://osf.io/2cb4q) prior to analysing data. Subsequent deviations from this preregistration are described in the supporting information (see supporting file 2).

##### 2.2.1.1. Characteristics of the PWTS Process

Management and implementation of the scheduling process was assessed through 23 items (see Items 1–18 and 23–27 in Table S1, supporting file 3), which asked respondents to rate their agreement (1, Totally Agree; 5, Totally Disagree; 6; Not Applicable for Me) with statements concerning the following:

• Clarity of expectations (e.g., I know what is expected of me in terms of the number of weekend shifts per schedule period).
• Active participation (e.g., I am active in the adjustment and equalisation process).
• Collaboration (e.g., In my department, we employees help each other to achieve the schedule based on our wishes and staffing needs).
• Fairness (e.g., In my department, the scheduling is imbued with justice).
• Responsibility for well‐being (e.g., I myself need to take responsibility for getting a schedule that I think is healthy).
• Leave distribution (e.g., I try to spread out my leave in the schedule).
• Scheduling support (e.g., My manager/the staffing assistant is open to discussing my working hours if I need to).
• Technical systems (e.g., The system is easy to use).

Four additional items (see Items 19–22 in Table S1, supporting file 1) concerning management and implementation asked respondents about:

• The importance that their manager attached to scheduling work hours to promote recovery (1, *very important* and 5, *not at all important*).
• The way in which they could influence their schedule (5 response options: e.g., I get to choose which shifts I want and can work during the schedule period; I have a fixed pattern that is copied at each schedule period in which I can make requests for changes).
• Whether they could veto aspects of a schedule they had been given (1, *yes*, *always*; 4, *no*, *never*; 5, *we do not use vetoes*).
• How many days’ notice they got of a new schedule (open response).

The importance that respondents placed on personal priorities when planning their schedules (1, *very important*; 5, *very unimportant*; 6, *not applicable*) was assessed with 10 items (see Items 28–37 in Table S1, supporting file 3). These covered family considerations, distribution of rest days, leisure activities, getting sufficient rest between shifts, the sleep rhythm, health, running personal errands, commuting, which colleagues worked those shifts and overtime compensation.

##### 2.2.1.2. Characteristics of the Work Environment

Perceived sufficiency of staffing levels (forthwith termed staffing sufficiency) was assessed with a single item (in my department, we have high enough staffing so that we employees can have healthy schedules. 1, *totally agree*; 5, *totally disagree*; 6, *not applicable for me*). Job tempo was assessed with a single item (Do you have to keep a high work pace? 1, *always*; 5, *never*/*almost never*).

Satisfaction at work was assessed with three items that assessed how often the respondent enjoyed their job (1, *very often or always*; 5, *very rarely or never*), their satisfaction with the possibility to influence their work hours (1, *very satisfied*; 5, *very dissatisfied*), and how they evaluated their work hours (1, *very bad*; 5; *very good*). A fourth item asked respondents to rate their agreement with the statement ‘I often think that I should leave my current workplace’ (1, *totally agree*; 5, *totally disagree*; 6, *not applicable for me*).

##### 2.2.1.3. Demographic Information

Demographic information included in the current analyses was obtained through six items: sex (woman, man, nonbinary, other alternative, uncertain, unwilling to answer), age, marital status (coresiding, in relationship but not coresiding, living alone), parental status (child, no child), job title (nurse/specialist nurse, assistant nurse, care assistant, mental health worker, other) and percentage of fulltime employment.

#### 2.2.2. Objective Working Time Data

In addition to the collection of survey data, each participant’s realised work hours were derived from the hospitals’ administrative systems for the 90‐day period that preceded that participant’s completion of the survey. The raw data included the unique ID numbers for each employee and the number of minutes worked each hour over every 24‐h period. It comprised records from ordinary shifts, overtime/extra shifts, shifts compensated by the hour and shifts where employees had been absent. Data from overtime/extra shifts and from shifts compensated by the hour were combined with data from the ordinary shifts to provide the total hours worked.

The objective working time variables used in the present study were defined and calculated following the procedures described by Harma et al. [9]. The raw data were transformed into clock start and end times for each worked shift. Overlapping shifts (e.g., an overtime shift overlapping an ordinary shift) were combined into one shift using the earliest start time and the latest end time. Where there were two registered shifts on one day, these were combined into one shift if there was an hour or less between them; otherwise, they were considered two separate shifts [19]. Shifts < 3 h were removed unless they were within an hour of another shift [20]. The shift data were then summarised into 9 parameters. Each parameter represented the frequency with which a working time characteristic occurred within the 90‐day reference period, calculated as a proportion. Each parameter was then dichotomised, with prevalence cut points of either > 25%, > 10% or > 0% representing the criteria for poor ergonomic schedules. These cut points were based on Harma et al. [21] and Karhula et al. [22], with modifications appropriate to common working time practises in Swedish healthcare (see Table 1).

**TABLE 1:** Definitions of objective working time variables.

Parameter description	Frequency calculated as proportion of	Cut point
Long weekly work hours (> 40 h)	All work weeks	> 25%
Very long working weeks (> 48 h)	All work weeks	> 10%
Long shifts (≥ 12 h)	All work shifts	> 0%
Night shifts	All work shifts	> 10%
Quick returns (< 11 h)	All work shifts	> 25%
Single days off	All days off	> 25%
> 4 consecutive work shifts	All spells of consecutive work shifts	> 25%
< 28‐h recovery periods after a last night shift	All recovery periods after a last night shift	> 0%
≥ 4 consecutive night shifts	All the spells of consecutive night shifts	> 0%

### 2.3. Statistical Analysis

The analysis plan was preregistered prior to analysis (https://osf.io/2cb4q). Subsequent deviations from this analysis plan are described in supporting file 2. Data analysis was conducted using SPSS Version 29 (IBM, 2023). The multiple imputation procedure within SPSS was used to impute values for missing data for the variables that were entered into the factor analysis (see below), with five imputed datasets obtained from the original.

Prior to the main analyses, the majority of items measuring the characteristics of PWTS and the work environment variables were reverse coded to aid ease of interpretation (excepting the items ‘I need more knowledge/training on how to use the system’, ‘I like to compress my work shifts to get longer leave’, ‘How do you like your working hours?’ and ‘I often think that I should leave my current workplace’). In order to maximise the sample available for factor analysis, items that included the response option ‘not applicable’ were recoded so that ‘not applicable’ was given the same value as ‘do not agree at all’ (in 23 of the 24 questions about the scheduling process) or ‘very unimportant’ ’ (in the 10 questions about the importance respondents placed on personal priorities). In the item about vetoes, response option ‘we don’t use vetoes’ ’ was given the same value as ‘never’. Table S1 details the number of responses recoded in this way. The effect of these recodings was that while high scores on these items indicated agreement with the statement/high importance/often getting vetoes, the lowest scores indicated an absence of agreement (i.e., either ‘disagree’ or ‘not applicable’)/absence of importance (i.e., either ‘not at all important’ or ‘not applicable’)/absence of vetoes (i.e., either ‘never’ or ‘we don’t use vetoes’). Therefore, care is needed when interpreting responses indicating an absence of agreement/importance/vetoes. The item asking how many days’ notice was given of new schedules was recoded into three categories: up to 7 days, 8–14 days and 15–90 days.

The initial stage of the analysis was to factor analyse the items assessing PWTS characteristics, to identify predictors for the second stage of the analysis. Kaiser–Meyer–Olkin statistics and Bartlett’s test of sphericity were deployed to check assumptions for EFA. While factors in these types of data cannot be assumed to be independent, the decision on whether to use orthogonal or oblique was based on comparisons of the intrafactor correlations obtained from orthogonal and oblique rotations, after Tabachnick and Fidell [23]. The factor scores were calculated as the means of the factor scores obtained from each of the five imputed datasets.

In the next stage of the analyses, a series of binary logistic regressions were conducted to examine whether the *PWTS characteristics* identified in the first stage predicted *working time characteristics*. We also examined whether *PWTS characteristics* predicted *satisfaction with work hours* (dichotomised into neutral/good and rather/very bad). In addition to these primary (regression) analyses, secondary (regression) analyses examined whether *PWTS characteristics* predicted three other indices of satisfaction, namely how often the respondent enjoyed of their job (dichotomised into sometimes/often/always and rarely/never); satisfaction with influence over work hours (dichotomised into neutral/satisfied and rather/very dissatisfied); and whether the respondent often thought of leaving their current workplace (dichotomised into neutral/disagree and agree). Each analysis adjusted for variables that could confound the associations of interest, namely, age (continuous variable), sex (dichotomised into female and male/other), marital status (living alone and other), parental status (parent and nonparent/no answer), job title (nurse/specialist nurse and auxiliary nurse/care assistant/other), work tempo (continuous variable) and staffing sufficiency (totally/somewhat agree/neutral and somewhat/totally disagree). Part‐time status (0%–75% and 76%–100%) was also included as an adjustment variable for each analysis, with the exceptions of the analyses of predictors of long weekly work hours (> 40 h) and very long weekly work hours (> 48 h).

Prior to each of the main regression analyses, a series of preliminary analyses were conducted to confirm: a linear relationship between the continuous predictors and the logit transform of the dependent variable (the Box–Tidwell approach); an absence of multicollinearity among the predictor variables and an absence of outliers in the solution [23].

For each regression, the main predictors (namely the factor scores associated with each of the PWTS characteristics identified) were entered in the first step (Model 1). Adjustment covariates were entered in the second step (Model 2). To control for multiple testing, Bonferroni familywise correction was applied within the two family sets of the analyses: Family 1—PWTS characteristics as predictors of working time characteristics (9 analyses) and Family 2—PWTS characteristics as predictors of satisfaction (4 analyses).

All 248 participants were included in the factor analysis. However, some of the participants were excluded from some of the regression analyses, as follows. Participants for whom objective working time data were unavailable (*N* = 10) were excluded from analyses of those outcome variables, leaving *N* = 238 in those analyses. Of the remainder, participants working part time (≤ 75%; *N* = 19) were excluded from the analyses of predictors of long weekly work hours (> 40 h) and very long weekly work hours (> 48 h), leaving *N* = 219 in those two analyses.

### 2.4. Ethical Considerations

This study was approved by the Swedish Ethical Review Authority (Dnr 2019‐05245). The study followed the Declaration of Helsinki regulations [24] and local ethical guidelines and regulations [25]. Prior to starting, the survey participants were assured that their responses would be kept confidential that their participation was voluntary and that they could withdraw at any time. Participants were then required to indicate their informed consent before beginning the survey.

## 3. Results

### 3.1. Study Participants

The mean age of the entire sample of respondents (*N* = 248) was 44.1 years (SD = 11.4); 91.9% were female; 21.4% lived alone; 73.8% were parents; 56% were nurses or specialist nurses, 36.3% were auxiliary nurses, 7.7% were care assistants; and 6.5% worked part time. Mean reported work tempo was 2.0 (SD = 0.6), while 81.9% reported staffing levels as being insufficient for employees to have healthy schedules.

### 3.2. Factor Analysis of the Items Assessing PWTS Characteristics

Of the 37 items initially identified for the factor analysis, 13 were identified as having extremely restricted distributions (> 75% responding ‘agree’ or ‘totally agree’) and were excluded (see Table S1). For the remaining 24 variables, multiple imputations of missing data were conducted (missing data were identified in 22 of 24 variables, 52% of cases, 5% of values. See Table S1). Variables with skewness outside −0.5 to 0.5 were subject to transformation; no variables were identified with kurtosis outside −2.0 to 2.0 (see Table 2 for the details of transformations).

**TABLE 2:** Mean factor loadings from 5 imputations of the final solution.

Item	SSPI	PPM	UST	SCP	PCH	BSW
I feel that my manager/the staffing assistant tries to accommodate my schedule wishes as much as possible^∗^	**0.81**	−0.16	0.08	0.24	0.01	0.04
My manager/the staffing assistant is open to discussing my working hours if I need to^∗^	**0.78**	−0.03	0.03	0.03	−0.08	0.08
My schedule wishes are met to a large extent	**0.68**	−0.15	0.08	0.26	0.18	−0.05
My manager/the staffing assistant knows and takes into account the possibilities and limitations I have for working different shifts and shift combinations	**0.65**	0.02	0.01	0.23	−0.02	0.11
My manager/the staffing assistant has good insight into my working hours	**0.63**	0.00	0.09	0.08	−0.02	0.05
If I need it, there is an opportunity to get support in scheduling^∗^	**0.48**	0.10	0.14	0.22	−0.11	0.09
How important do you think your manager thinks it is with working hours that enable recovery?	**0.44**	0.11	−0.16	−0.05	0.08	0.34
Do you usually get through the vetoes you put?	0.27	−0.15	0.11	0.24	0.09	0.14
When planning and adjusting your schedule, how important is it to you to consider the following factors?						
… To receive overtime compensation	0.03	**0.72**	0.10	0.11	−0.10	−0.12
… Which other employees work those shifts	−0.05	**0.57**	0.04	0.01	−0.01	0.00
… Being able to run errands (including, e.g., doctor’s appointments)^∗^	0.04	**0.56**	0.05	0.01	−0.05	0.12
… Your ability to commute to and from work	−0.01	**0.55**	−0.01	−0.06	−0.01	0.11
How far in advance are you typically told what your set schedule will look like for a new schedule period?	−0.07	0.28	−0.05	−0.02	0.04	−0.17
The system is easy to use #	0.04	0.02	**0.70**	0.04	0.05	0.03
I think the technical systems help me get an overview of the schedule #	0.05	0.12	**0.67**	0.22	0.10	0.33
I need more knowledge/training on how to use the system^°^	0.08	0.03	**0.46**	−0.03	−0.12	−0.22
In my department, we employees help each other to achieve the schedule based on our wishes and staffing needs	0.19	0.01	0.12	**0.65**	0.06	0.02
In my department, the scheduling is imbued with justice	0.39	0.03	0.13	**0.43**	0.00	0.17
I usually base my schedule on staffing needs	0.25	0.03	−0.06	**0.42**	−0.02	0.05
I think it is easy to get an overall picture of the schedule when I am scheduling my own shifts^∗^	0.23	−0.01	0.39	**0.41**	0.05	0.31
I try to spread out my leave in the schedule	0.01	0.10	0.02	0.28	**0.74**	0.01
I like to compress my work shifts to get longer leave^∗^	0.02	0.17	0.01	0.14	**−0.52**	0.02
My manager/the staffing assistant points out if there is too little recovery in the schedule that I wanted^°^	−0.39	−0.10	0.16	−0.01	−0.05	**−0.48**
The system warns of bad shift combinations	0.06	−0.04	0.12	0.18	−0.07	**0.37**

*Note:* Items marked ^∗^ were subject reflection square root transformation, prior to being entered into the analysis. Items marked # were subject logarithmic transformation, prior to being entered into the analysis. Items marked ^°^ were subject reflection and logarithmic transformation, prior to being entered into the analysis. Bold values indicate the primary factor loadings for each item.

Abbreviations: BSW = bad schedule warnings, PCH = preference for compressing hours, PPM = prioritising personal motives, SCP = scheduling as a collective process, SSPI = scheduling support & personal influence, UST = usability of scheduling tools.

EFA indicated the presence of 6 factors (see Table 2). Comparisons of orthogonal and oblique rotations indicated that intrafactor correlations when using oblique rotation were low (< 0.3), and thus, the decision was taken to employ orthogonal (varimax) rotation. KMO values for the five imputations ranged between 0.798 and 0.799, and Bartlett’s test of sphericity between *χ* ^2^ = 1615.521, *p* < 0.001 and 1647.543, *p* < 0.001, indicating that the assumption for EFA was met. Correlations among the six factors ranged between 0.102 and −0.026.

Seven items loaded on the first factor, which was labelled *Scheduling Support & Personal Influence* (SSPI), with factor loadings ranging from 0.44 to 0.81. The item ‘How important do you think your manager thinks it is with working hours that enable recovery?’ cross‐loaded onto the sixth factor.

Four items loaded onto the second factor, which was labelled *Prioritising Personal Motives* (PPM), with factor loadings ranging between 0.55 and 0.72.

Three items loaded onto the third factor, which was labelled *Usability of Scheduling Tools* (UST), with loadings ranging from 0.46 to 0.70. The item ‘I think the technical systems help me get an overview of the schedule’ cross‐loaded onto the sixth factor. The item ‘I need more knowledge/training on how to use the system’ was subject to reflection transformation, reversing its polarity; however, as the polarity of the item was opposite to the other items (i.e., it was one of the items that was not reverse coded at the outset), it appears with a positive factor loading in Table 2.

Four items loaded onto the fourth factor, which was labelled *Scheduling as a Collective Process* (SCP), with factor loadings of 0.40–0.66. The item ‘In my department, the scheduling is imbued with justice’ cross‐loaded onto the first factor, and the item ‘I think it is easy to get an overall picture of the schedule when I have to enter my own shifts’ cross‐loaded onto the first and sixth factors.

Two items loaded onto the fifth factor, which was labelled *Preference for Compressing Hours* (PCH), with factor loadings of −0.52 and 0.74. The item ‘I like to compress my work shifts to get longer leave’ has an opposite polarity to the other items (i.e., it was one of the items that was not reverse coded at the outset) and so it appears with a negative factor loading in Table 2.

Two items loaded onto the sixth factor, which was labelled ‘*Bad Schedule Warnings*’ (BSW), with factor loadings of −0.48 and 0.37. The item ‘My manager/the staffing assistant points out if there is too little recovery in the schedule that I wanted’ cross‐loaded onto the first factor. This item was subject to reflection transformation, reversing its polarity such that it was given a negative factor loading.

Two items loaded below 0.3 on all four factors (‘Do you usually get through the vetoes you put?’ and ‘How far in advance are you typically told what your set schedule will look like for a new schedule period?’), indicating that the items were not making significant contributions to any of the factors. Thus, of the 24 items included in the factor analysis, 22 contributed to the final set of abstracted factors.

### 3.3. Regression of PWTS Characteristics on Working Time Characteristics and Satisfaction Indices

#### 3.3.1. Primary (Regression) Analyses: PWTS Characteristics as Predictors of Working Time Characteristics and Satisfaction With Work Hours

Table 3 details the distribution of the outcome variables, as well as means and SDs of the main predictor variables by outcome category.

**TABLE 3:** Distributions of outcomes (%); PWTS characteristics (mean and SD) by outcome categories.

		%	SSPI		PPM		UST		SCP		PCH		BSW	
			Mean	SD	Mean	SD	Mean	SD	Mean	SD	Mean	SD	Mean	SD
Long weekly work hours (> 40 h)	≤ 25%	38.7	0.09	0.99	−0.08	0.89	0.13	0.86	0.25	0.58	0.17	0.79	−0.01	0.68
	> 25%	61.3	−0.05	0.87	0.05	0.84	−0.08	0.83	−0.15	0.85	−0.12	0.80	−0.01	0.79

Very long working weeks (> 48 h)	≤ 10%	61.3	0.02	0.92	−0.10	0.86	0.03	0.88	0.12	0.71	0.03	0.86	−0.05	0.70
	> 10%	38.7	−0.02	0.94	0.16	0.85	−0.05	0.79	−0.17	0.86	−0.06	0.72	0.05	0.83

Long shifts (≥ 12 h)	0%	71.8	0.01	0.93	−0.02	0.87	0.00	0.86	0.08	0.76	0.08	0.81	−0.09	0.73
	> 0%	28.2	−0.01	0.91	0.05	0.84	0.01	0.82	−0.18	0.82	−0.22	0.76	0.20	0.78

Night shifts	≤ 10%	21.4	0.14	0.97	0.07	0.90	0.09	0.83	0.20	0.76	0.16	0.85	−0.08	0.74
	> 10%	78.6	−0.03	0.91	−0.02	0.85	−0.02	0.85	−0.05	0.78	−0.05	0.79	0.01	0.75

Quick returns (< 11 h)	≤ 25%	93.3	0.02	0.93	0.00	0.85	−0.02	0.84	0.00	0.79	0.00	0.80	0.00	0.76
	> 25%	6.7	−0.18	0.87	0.01	0.98	0.34	0.91	0.07	0.76	−0.13	0.96	−0.19	0.62

Single days off	≤ 25%	77.3	−0.02	0.95	−0.01	0.86	0.04	0.85	0.02	0.78	−0.03	0.83	−0.05	0.74
	> 25%	22.7	0.08	0.81	0.03	0.88	−0.15	0.81	−0.05	0.81	0.06	0.72	0.15	0.76

> 4 consecutive work shifts	≤ 25%	88.7	0.01	0.91	0.00	0.86	0.01	0.83	0.04	0.73	0.00	0.80	−0.01	0.74
	> 25%	11.3	−0.03	1.02	−0.03	0.92	−0.10	0.97	−0.31	1.07	−0.08	0.88	−0.02	0.86

< 28‐h recovery periods after a last night shift	0%	68.9	−0.08	0.91	−0.02	0.89	0.02	0.88	0.01	0.79	−0.03	0.84	−0.01	0.76
	> 0%	31.1	0.18	0.92	0.04	0.80	−0.03	0.76	−0.01	0.76	0.05	0.75	0.00	0.73

≥ 4 consecutive night shifts	0%	82.8	0.05	0.93	−0.05	0.86	0.02	0.86	0.04	0.74	0.04	0.82	−0.03	0.74
	> 0%	17.2	−0.23	0.84	0.21	0.82	−0.09	0.78	−0.16	0.97	−0.21	0.73	0.11	0.80

Like work hours	Neutral/good	82.3	0.12	0.91	0.00	0.86	0.02	0.84	0.00	0.81	0.00	0.82	0.02	0.74
	Bad	17.7	−0.42	0.88	0.01	0.89	−0.07	0.88	−0.01	0.66	0.01	0.77	−0.07	0.74

Enjoy my job	Sometimes/often	79.4	0.03	0.93	0.01	0.87	0.02	0.83	0.07	0.79	0.01	0.80	−0.04	0.75
	Rarely	20.6	−0.10	0.90	−0.04	0.85	−0.08	0.94	−0.27	0.71	−0.02	0.85	0.14	0.70

Satisfied with influence over work hours	Satisfied/neutral	78.2	0.09	0.90	0.05	0.88	0.02	0.85	0.03	0.78	0.04	0.79	0.04	0.77
	Dissatisfied	21.8	−0.43	0.93	−0.21	0.73	−0.09	0.82	−0.15	0.78	−0.18	0.91	−0.16	0.60

Thinking of leaving	Disagree/neutral	71.8	0.16	0.84	0.01	0.82	−0.02	0.83	0.00	0.82	−0.04	0.84	0.03	0.76
	Agree	28.2	−0.42	1.00	−0.02	0.98	0.06	0.89	0.01	0.69	0.11	0.72	−0.07	0.71

Abbreviations: BSW = bad schedule warnings, PCH = preference for compressing hours, PPM = prioritising personal motives, SCP = scheduling as a collective process, SSPI = scheduling support & personal influence, UST = usability of scheduling tools.

In the analysis of predictors of long weekly work hours (> 40 h), SCP (OR = 1.89; 95% CI = 1.24, 2.86; *p* < 0.05) and PCH (OR = 1.74; 95% CI = 1.19, 2.54; *p* < 0.05) were both significantly associated with exceeding the cut‐off for excessive long weekly work hours in Model 1, after applying the Bonferroni correction. The associations remained significant after adjustment (Model 2. OR = 2.04; 95% CI = 1.31, 3.17; *p* < 0.05 and OR = 1.76; 95% CI = 1.19, 2.60; *p* < 0.05). These results indicated that long weekly work hours were more likely when respondents (1) perceived SCP and (2) preferred to compress their work shifts in order to get longer leave (as opposed to spreading out their leave in the schedule). See Table S2 for a full description of the results. In the analyses of very long weekly work hours (> 48 h), none of the variables were identified as significant predictors, after applying the Bonferroni correction. See Table S3 for a full description of the results.

In the analysis of predictors of long shifts (≥ 12 h), BSW (OR = 0.54; 95% CI = 0.36, 0.82; *p* < 0.05) was significantly associated with exceeding the cut‐off in Model 1, after applying the Bonferroni correction. The association remained significant after adjustment (Model 2. OR = 0.53; 95% CI = 0.34, 0.82; *p* < 0.05). The result indicated that respondents who reported being alerted to bad schedules by the system or by their manager were less likely to work long shifts. See Table S4 for a full description of the results.

In the analyses of the other working time characteristics, none of the variables were identified as significant predictors, after applying the Bonferroni correction. See Tables S5–S10 for full descriptions of the results.

In the analyses of predictors of satisfaction with work hours, high scores on SSPI were associated with higher satisfaction with work hours in the unadjusted analysis (Model 1. OR = 1.93; 95% CI = 1.34, 2.78, *p* < 0.01). The association remained significant after adjustment (Model 2. OR = 1.92; 95% CI = 1.30, 2.82, *p* < 0.01). See Table S11 for a full description of the results.

#### 3.3.2. Secondary (Regression) Analyses: PWTS Characteristics as Predictors of Job Satisfaction, Satisfaction With Influence Over Work Hours and Thoughts of Leaving the Workplace

In the analysis of predictors of enjoying the job (job satisfaction), none of the variables were identified as significant predictors, after applying the Bonferroni correction. See Table S12 for a full description of the results.

In the analysis of predictors of satisfaction with influence over work hours, high scores on SSPI were associated with more positive evaluations of influence over work hours in the unadjusted analysis (Model 1. OR = 2.25; 95% CI = 1.45, 3.49; *p* < 0.001). The association remained significant after adjustment (Model 2. OR = 2.04; 95% CI = 1.29, 3.22, *p* < 0.01). See Table S13 for a full description of the results.

In the analysis of predictors of thinking about leaving the workplace, high scores on SSPI predicted disagreement (i.e., high scores) with the statement ‘I often think that I should leave my current workplace’; i.e., higher SSPI predicted a lower likelihood of thinking about leaving (Model 1. OR = 2.06; 95% CI = 1.47, 2.90; *p* < 0.001). The association remained significant after adjustment (Model 2. OR = 2.24; 95% CI = 1.54, 3.28; *p* < 0.001). See Table S14 for a full description of the results.

## 4. Discussion

This study sought to characterise the different conditions under which PWTS was implemented, and the different approaches taken by employees and managers to that implementation, at 12 units within two Swedish hospitals. Six parameters of PWTS characteristics were identified, namely SSPI, PPM, UST, SCP, PCH and BSW. The study further sought to determine whether differences in these PWTS characteristics predicted the ergonomic characteristics of the schedules that emerged from the PWTS process and employee satisfaction. In the primary (regression) analyses, associations were observed between PWTS characteristics and frequency of long weekly work hours (> 40 h per week), frequency of long shifts (≥ 12 h) and satisfaction with work hours. In addition, secondary (regression) analyses identified associations between PWTS characteristics and satisfaction with influence over work hours and with thinking about leaving the current workplace.

Analyses of working time characteristics found that respondents were more likely to exceed the criteria for excessive long work weeks (> 25% of all work weeks > 40 h) if they preferred to compress their work hours, to get longer but less frequent periods of time off (factor PCH). PWTS has previously been found to be associated with higher frequency of long work weeks, when compared to traditional scheduling [26]. The current finding supports the interpretation offered in that study, in which higher frequency of long work weeks in PWTS settings reflects employees’ desire to compress their work hours, so as to obtain longer periods of time off between work weeks. Preference for compressing work hours did not, however, predict very long work weeks (> 48 h). This suggests that while some nurses and/or their managers deemed moderately long working weeks to be acceptable (e.g., to get longer periods of time off), the negative consequences of working very long weeks (e.g., greater fatigue and work–family interference) were considered too high a price to pay. Employees using PWTS can also compress their work hours by choosing to work long shifts [3, 8], although we found no association between the preference for compressing shift sequences to get longer leave and working long shifts (≥ 12 h).

Respondents were also more likely to work long work weeks (> 40 h) if they scored higher on the factor SCP. This factor is interpreted as the extent to which the PWTS process is perceived as a collective effort that seeks to optimise schedules for all stakeholders. Of the six factors identified in the current EFA, this PWTS characteristic most clearly distinguishes PWTS, as a collaborative approach to schedule planning [3], from other more individualistic forms of self‐scheduling [12]. Anecdotal evidence from our interviews leads us to suggest that a collective sense of collaboration and mutual support within teams may make it easier for those who wish to work longer hours to do so. It also suggests that such an environment may promote a sense of ‘give and take’, with individuals being more willing to work long hours when needed if they sense that, by supporting their team in this way, they can expect to have their own needs and preferences met when the time comes. Further research (e.g., comparing PWTS with more individualistic forms of self‐scheduling) is needed to verify these suggestions.

Respondents who more commonly reported being alerted to bad schedules by the scheduling system or by their manager (factor BSW) were less likely to work long shifts (≥ 12 h). While this might suggest that such warnings are deterring the scheduling of long shifts, it is also conceivable that warnings are *less* likely to be given by managers in work settings where long shifts are *more* common, e.g., because long shifts are deemed to be an operational necessity.

Concerns have been raised that if workers are allowed to influence their work hours, there is risk that they will choose ergonomically unsound schedules over ones that promote better health and recovery [27]. However, previous research has found that enhanced WTC need not result in workers choosing ergonomically unsound schedules [8, 22]. Similarly, previous studies comparing PWTS with traditional scheduling arrangements have found that, with the exception of length of work hours, working time characteristics are not much influenced by which type of scheduling process is practised [3, 26]. Building on this, the current results indicated that working time characteristics differed little between different forms of PWTS (i.e., ones that differed with respect to the conditions of implementation, and the approaches taken by employees and staff to that implementation). In particular, we found no evidence that ergonomic thresholds were more likely to be breached by respondents who prioritised their personal motives when choosing schedules (factor PPM).

Higher scores on factor SSPI were identified as a significant predictor of higher employee satisfaction with work hours and, in the secondary (regression) analyses, of higher satisfaction with influence over work hours, as well as fewer thoughts about leaving the workplace. The findings are consistent with previous research demonstrating the importance that influence over work hours has for maintaining employee satisfaction [28] and reducing turnover intention [29]. They also reaffirm our earlier qualitative findings [18] and findings elsewhere [14], highlighting the important role played by first‐line managers in the successful implementation of PWTS. Sustainable work hours are promoted by managers who foster individual relationships with employees, maintaining an open dialogue that gives insight into the individual employee’s life circumstances, preferences and tolerance for shifts and shift combinations [18]. Previous research has also shown that having managers who actively support employees’ influence over their work hours contributes to the maintenance of low levels of sick leave [30]. That study also highlighted how managers may find themselves challenged to facilitate flexibility in the face of demands from senior management, e.g., in relation to budgetary constraints. This suggests a need for future research to explore how first‐line managers can be supported in their efforts to promote sustainable work hours.

Despite our earlier finding from interviews that technological systems used in the scheduling process were instrumental in the creation of sustainable schedules [18], the current results provided no indication that the usability of these systems (factor UST) impacted on satisfaction with the schedules that were produced. As noted in those earlier interviews, the potential benefits of these systems may be diminished if the warnings are difficult to understand, which may account for this null finding. Nevertheless, as already discussed, there was evidence from the objective working time data of the potential value of warnings; participants who reported being alerted to bad schedules by the scheduling system and/or by their manager were less likely to work long shifts (≥ 12 h). Thus, it seems likely that well‐designed scheduling software has a role to play in the creation of sustainable schedules, as do leaders who actively highlight the risks of ergonomically unsound schedules.

Our earlier qualitative study [18] highlighted the perceived importance of establishing a shared responsibility framework (i.e., active engagement by all persons involved in the scheduling process) and of fairness (e.g., in the distribution of unpopular shifts). On this basis, it could have been anticipated that factor SCP would have predicted satisfaction outcomes in the current analyses, although this turned out not to be the case.

In our previous research, it was reported by managers and scheduling assistants that understaffing was a barrier to achieving sustainable work hours in the context of PWTS [18]. Thus, it could be anticipated that sustainable work hours may be difficult to achieve when staffing is insufficient, irrespective of how PWTS is being implemented. This would suggest that PWTS characteristics would be less likely to predict indices of sustainable work hours (i.e., work hours characteristics and satisfaction) when staffing levels were insufficient. The analysis plan for the current study included an investigation of whether staffing sufficiency would moderate associations between PWTS characteristics and the measured outcomes. However, this aspect of the analysis plan could not be realised due to the small proportion of respondents reporting sufficient staffing, resulting in insufficient statistical power to conduct moderation analyses. Future studies should consider the potential moderating effects of staffing sufficiency on associations between PWTS characteristics and indices reflecting the sustainability of work hours.

### 4.1. Strengths and Limitations

A key strength of the current study was the use of objective working time data to accurately capture the characteristics of the respondents’ work schedules, using a previously validated method of determining realised work hours [9]. Self‐reported estimations of hours worked and other schedule characteristics are prone to recall bias. However, the remainder of the measures were self‐report and thus subject to bias. Other limitations include the use of single‐item measures of work environment variables, including the measures of satisfaction and thoughts of leaving, which have not been externally validated. In order to maximise the sample available for analysis, it was necessary to collapse response categories for some items, reducing the interpretability of responses to those items. The sample was relatively small, and thus, null findings could be due to insufficient statistical power. The response rate was low, which could introduce bias: For example, it may be that staff who held the strongest opinions about PWTS were more likely to participate, thereby potentially enhancing the strength of associations between PWTS characteristics and ratings related to satisfaction. It was not possible to compare the objective hours data of responders to the survey with data of nonresponders, as we lacked the consent of nonresponders to access and analyse their work hour data. We lacked suitable information at the ward level to include covariates that varied systematically by ward. The study was conducted within two hospitals in two regions of Sweden, which may limit the generalisability of the findings, most especially beyond the context of Swedish healthcare settings. The impact of PWTS on outcomes such as satisfaction may vary between different occupational settings, depending on which parameters are flexible and which are fixed (e.g., in healthcare, even when self‐scheduling is practised, the start and end times of shifts are likely to remain fixed [3]). Lastly, causal interpretation of the observed associations is limited due to the cross‐sectional nature of the study design.

### 4.2. Implications for Nursing Management

The current findings have some key implications for the use of PWTS in nursing management practice. They indicate that first‐line managers in healthcare should prioritise giving influence over work hours in order to promote staff satisfaction, while at the same time providing support to staff in the scheduling process. Managers should seek to identify staff who choose to work longer weeks in order to compress their work weeks and bring to their attention the potential risks to both the employee’s own well‐being and to patient safety. Managers will be helped in this identification by having usable scheduling software that warns of ergonomically unsound schedules.

## 5. Conclusions

The current study found that PWTS characteristics were more consistently associated with indices of staff satisfaction than with objective ergonomic schedule characteristics. Sustainable work hours are characterised by the ergonomic qualities (or ‘soundness’) of the work schedules. In this regard, we found only limited evidence that differences in the approach to scheduling (i.e., variations in PWTS characteristics) affected the ergonomic characteristics of the schedules that emerged from this process. However, an important consequence of having sustainable work hours is that nurses feel satisfied with their work hours such that those work hours do not prompt the nurse to think about leaving their job. The current findings suggest that, for PWTS to be perceived positively, there should be open communication between employees and managers/staffing assistants, fostering individual relationships with employees and promoting employee voice in the scheduling process such that nurses feel able to influence their work hours. Overall, the current findings suggest that, while PWTS characteristics may contribute to the creation of sustainable work schedules, other factors (e.g., stakeholders’ awareness of what constitutes healthy work hours; resources) will also play an important role.

## Author Contributions

Philip Tucker was responsible for the conceptualisation of the study, data analysis and writing the first draft of the manuscript. Majken Epstein was responsible for the conceptualisation of the study, data collection and revising the manuscript. Anna Dahlgren was responsible for overall project management, conceptualisation of the study, funding acquisition and revising the manuscript.

## Funding

This study was funded by Afa Försäkring (No. 180242).

## Disclosure

The funders had no role or influence on the research process or outcomes. The funders played no role in any part of the production of the current paper; the study design, data collection, analysis and interpretation of the data; writing of the report and decision to publish or preparation of the manuscript.

## Conflicts of Interest

The authors declare no conflicts of interest.

## Supporting Information

Additional supporting information can be found online in the Supporting Information section.

## Supporting information


**Supporting Information 1** File 1: STROBE checklist.

**Supporting Information 2** File 2: Deviations from preregistration.

**Supporting Information 3** File 3: Tables S1–S14.

## Data Availability

The data that support the findings of this study are available on request from the corresponding author. The data are not publicly available due to privacy or ethical restrictions.
