# Supplementary material for: Promoting Sustainable Work Hours Through Participatory Scheduling: A Cross‐Sectional Study Employing Objective Working Time Data
Source: J Nurs Manag. 2026 Aug 17;2026:4164713. doi: 10.1155/jonm/4164713 (PMC13478799; doi:10.1155/jonm/4164713)
Supplement: Supplementary file 1 [file JONM-2026-4164713-s002.docx]

**SUPPLEMENTARY MATERIAL 2**

**Deviations from pre-registration (original at** [**https://osf.io/2cb4q**](https://osf.io/2cb4q)**)**

1. **Summary and aims**

The analysis plan for the current study included an investigation of whether staffing sufficiency would moderate associations between PWTS characteristics and the measured outcomes. However, this aspect of the analysis plan could not be realised due to the small proportion of respondents reporting sufficient staffing, resulting in insufficient statistical power to conduct moderation analyses.

The analysis plan also included exploratory analyses to explore the relationships between realised work hours in an PWTS setting and satisfaction, wellbeing and cognitive function; and whether these associations are moderated the ‘fit’ - whether there is a match between preferred and actual work hours. With the exception of two measures related to satisfaction (satisfaction with influence over work hours and thinking about leaving the workplace) the exploratory analyses failed to produce any significant effects. The final manuscript only reports the exploratory analyses of the main effects of three variables related to satisfaction (‘satisfaction with influence over work hours’, ‘thinking about leaving the workplace’ and ‘enjoying the job’), as well as ‘satisfaction with work hours’ (as per the planned main analysis). As stated in the analysis plan, “All exploratory analyses may not be reported in the final manuscript, but if they are reported they will be classified as exploratory”.

**2	Study information**

**2.2	Main analysis**

As noted above, examination of staffing levels as a potential moderator was dropped (Research Question 1b).

**2.3	Exploratory analysis**

As noted above, only the main effects of the three variables related to satisfaction were reported in the exploratory analyses.

**3	Variables**

**3.1	Main analysis**

***3.1.1	Independent variables***

Of the 37 items initially identified for the factor analysis, 13 were identified as having extremely restricted distributions (> 75% responding ‘agree’ or ‘totally agree’) and were excluded.

***3.1.2	Moderator variable***

As noted above, staffing levels was not examined as a moderator.

***3.1.3.1	Working time characteristics***

The following variable was not calculated:

*% of double shifts (≥15 hours) of all the work shifts (DoubleShifts_%)*

***3.1.4	Covariates***

The following variables that were included as covariates in the original analysis plan were excluded due to issues of multicollinearity:

- Job experience *yrkeserfarenhet*
- Job control *Arb_påverka*
- Job support *Arb_samarb*

The variable ‘Percentage of fulltime (*heltid_deltid)’* was included as an adjustment variable for each analysis, with the exceptions of the analyses of predictors of long weekly work hours (> 40 hours) and very long weekly work hours (> 48 hours).

Staffing level, which had been planned to be analysed as a potential moderating factor (see above) was added as an additional covariate in each analysis.

**3.2	Exploratory analyses**

3.2.2 **Moderator variable**

As noted above, exploratory analysis of the moderating effects of fit were excluded from the final manuscript.

**3.2.3	Dependent variables**

Of the dependent variables listed in the analysis plan, the manuscript report the analyses of main effects of the following variables:

- Satisfaction with working hours (*trivsel_arbtid)*
- Enjoyment of the job (*trivselarbete*)
- Thinking about leaving the workplace (*lämna_arbplats*)
- Satisfaction with influence over work hours (*nöjd_kontroll_arbtid*). Note that this variable was accidentally omitted from the original analysis plan.

**4	Analysis plan**

**4.1	Main analyses:**

***4.1.1	RQ1a and RQ1b***

As noted above, analyses were conducted on 9 dependent variables and did not include analyses of interactions between *PWTS characteristics* & *staffing level.*

**4.2	Exploratory analysis:**

As noted above, exploratory analyses were only reported for the main effects of three variables related to satisfaction.

***4.4.3.	Covariates***

Children (*barn)* was dichotomised as planned, but with null responses (no answer) recoded as equivalent to non-parent.

Job title (*yrkestitel)* was dischotomised as ‘nurse/specialist nurse’ and ‘auxiliary nurse/care assistant/other’).

Percentage of fulltime (*heltid_deltid*), renamed Part-time status, was dichotomised into 0-75%, and 76-100%.

***4.4.4.	Fit***

As noted above, analyses of this variable were not reported.

**4.5	Missing data**

The original analysis plan was to exclude missing data on a per-analysis basis. However, this would have resulted in unacceptably small sample sizes. In order to maximize the sample size for the exploratory factor analysis, it was decided to impute missing values using the multiple imputation procedure within SPSS.
