# Supplementary material for: Promoting Sustainable Work Hours Through Participatory Scheduling: A Cross‐Sectional Study Employing Objective Working Time Data
Source: J Nurs Manag. 2026 Aug 17;2026:4164713. doi: 10.1155/jonm/4164713 (PMC13478799; doi:10.1155/jonm/4164713)
Supplement: Supplementary file 2 [file JONM-2026-4164713-s003.docx]

Table S1: Items initially intended for factor analysis, exclusions, missing data and number of recoded responses.

|  | *Excluded from factor analysis due to restricted distribution?* | *Missing values* | | *Items recoded from 'Not applicable' etc. to 'Do not agree at all'* | |
| --- | --- | --- | --- | --- | --- |
| *Item* |  | *N* | *%* | *N* | *%* |
| **Below are a series of statements about the scheduling process. Indicate whether or not you agree with these. -** |  |  |  |  |  |
| 1. I know what is expected of me in terms of the number of weekend shifts per schedule period | Yes | 0 | 0 | n/a | n/a |
| 2. I know what is expected of me in terms of the number of evening shifts per schedule period | Yes | 3 | 1.2 | n/a | n/a |
| 3. I am active in the adjustment process/equalization process | Yes | 2 | 0.8 | n/a | n/a |
| 4. I think it is easy to get an overall picture of the schedule when I am scheduling my own shifts |  | 0 | 0 | 8 | 3.2 |
| 5. I usually base my schedule on staffing needs |  | 0 | 0 | 9 | 3.6 |
| 6. In my department, we employees help each other to achieve the schedule based on our wishes and staffing needs |  | 2 | 0.8 | 14 | 5.6 |
| 7. If I need it, there is an opportunity to get support in scheduling |  |  |  | 17 | 6.9 |
| 8. In my department, the scheduling is imbued with justice |  | 3 | 1.2 | 12 | 4.8 |
| 9. I myself need to take responsibility for getting a schedule that I think is healthy | Yes | 0 | 0 | n/a | n/a |
| 10. I feel I have enough knowledge to plan a schedule with adequate recovery | Yes | 1 | 0.4 | n/a | n/a |
| 11. I like to compress my work shifts to get longer leave |  | 1 | 0.4 | 13 | 5.2 |
| 12. I try to spread out my leave in the schedule |  | 2 | 0.8 | 9 | 3.6 |
| 13. My schedule wishes are met to a large extent |  | 1 | 0.4 | 9 | 36 |
| 14. I feel that my manager/staffing assistant tries to accommodate my schedule wishes as much as possible |  | 1 | 0.4 | 8 | 3.2 |
| 15. My manager/staffing assistant knows and takes into account the possibilities and limitations I have for working different shifts and shift combinations |  | 2 | 0.8 | 19 | 7.7 |
| 16. My manager/staffing assistant has good insight into my working hours |  | 0 | 0 | 8 | 3.2 |
| 17. My manager/staffing assistant is open to discussing my working hours if I need to |  | 1 | 0.4 | 6 | 2.4 |
| 18. My manager/staffing assistant points out if there is too little recovery in the schedule that I wanted |  | 1 | 0.4 | 28 | 11.3 |
| 19. How important do you think your boss thinks it is with working hours that enable recovery? |  | 0 | 0 | 0 | 0 |
| 20. In what way do you have influence over your schedule before a new schedule period? | Yes | 1 | 0.4 | n/a | n/a |
| 21. Do you usually get through the vetoes you put? |  | 2 | 0.8 | 3 | 1.2 |
| 22. How far in advance are you typically told what your set schedule will look like for a new schedule period? Enter approximate number of days. |  | 10 | 4 | n/a | n/a |
|  |  |  |  |  |  |
| **Below follows a series of statements about the technical systems used in scheduling -** |  |  |  |  |  |
| 23. I put my schedule in the system myself | Yes | 1 | 0.4 | n/a | n/a |
| 24. I think the technical systems help me get an overview of the schedule |  | 1 | 0.4 | 5 | 2 |
| 25. The system warns of bad shift combinations |  | 2 | 0.8 | 12 | 4.8 |
| 26. I need more knowledge/training on how to use the system |  | 0 | 0 | 15 | 6 |
| 27. The system is easy to use |  | 0 | 0 | 4 | 1.6 |
|  |  |  |  |  |  |
| **When planning and adjusting your schedule, how important is it to you to consider the following factors? -** |  |  |  |  |  |
| 28. Family life (e.g. partner, children, elderly family members) | Yes | 1 | 0.4 | n/a | n/a |
| 29. To get consecutive leave | Yes | 1 | 0.4 | n/a | n/a |
| 30. Leisure activities (e.g. friends, activities) | Yes | 1 | 0.4 | n/a | n/a |
| 31. Getting sufficient rest between work shifts | Yes | 1 | 0.4 | n/a | n/a |
| 32. Your sleep rhythm | Yes | 1 | 0.4 | n/a | n/a |
| 33. Your health | Yes | 0 | 0 | n/a | n/a |
| 34. Being able to run errands (including e.g. doctor's appointments) |  | 0 | 0 | 0 | 0 |
| 35. Your ability to commute to and from work |  | 2 | 0.8 | 43 | 17.3 |
| 36. Which other employees work those shifts |  | 0 | 0 | 16 | 6.5 |
| 37. To receive overtime compensation |  | 1 | 0.4 | 9 | 3.6 |

Table S2. Summary of hierarchical logistic regression analysis for variables predicting long weekly work hours (> 40 hours) (N = 219)

|  | Model 1 (Unadjusted) | | | | | Model 2 (Adjusted) | | | | |
| --- | --- | --- | --- | --- | --- | --- | --- | --- | --- | --- |
| Variable | *B* | *SE* | *Exp(B)* | *Lower* | *Upper* | *B* | *SE* | *Exp(B)* | *Lower* | *Upper* |
| SSPI | 0.17 | 0.16 | 1.19 | 0.86 | 1.63 | 0.14 | 0.17 | 1.15 | 0.82 | 1.60 |
| PPM | -0.22 | 0.17 | 0.81 | 0.58 | 1.13 | -0.13 | 0.19 | 0.88 | 0.61 | 1.26 |
| UST | 0.23 | 0.18 | 1.26 | 0.89 | 1.78 | 0.26 | 0.18 | 1.29 | 0.90 | 1.85 |
| SCP | 0.63 | 0.21 | **1.89*** | 1.24 | 2.86 | 0.71 | 0.23 | **2.04*** | 1.31 | 3.17 |
| PCH | 0.55 | 0.19 | **1.74*** | 1.19 | 2.54 | 0.57 | 0.20 | **1.76*** | 1.19 | 2.60 |
| BSW | -0.18 | 0.20 | 0.84 | 0.57 | 1.25 | -0.26 | 0.22 | 0.77 | 0.50 | 1.17 |
| Sex |  |  |  |  |  | 0.51 | 0.58 | 1.66 | 0.54 | 5.14 |
| Age |  |  |  |  |  | 0.01 | 0.02 | 1.01 | 0.97 | 1.04 |
| Marital |  |  |  |  |  | -0.19 | 0.38 | 0.83 | 0.39 | 1.75 |
| Children |  |  |  |  |  | 0.10 | 0.38 | 1.10 | 0.53 | 2.30 |
| Job title |  |  |  |  |  | 0.46 | 0.34 | 1.59 | 0.82 | 3.07 |
| Work tempo |  |  |  |  |  | -0.43 | 0.29 | 0.65 | 0.37 | 1.14 |
| Staffing |  |  |  |  |  | -0.08 | 0.41 | 0.93 | 0.41 | 2.08 |

*Note: SSPI = Scheduling Support & Personal Influence; PPM = Prioritising Personal Motives; UST = Usability of Scheduling Tools; SCP = Scheduling as a Collective Process; PCH = Preference for Compressing Hours; BSW = Bad Schedule Warnings.*

** = p < .05; ** = p < .01; *** = p < .001, after Bonferroni correction*

Table S3. Summary of hierarchical logistic regression analysis for variables predicting very long weekly work hours (> 48 hours) (N = 219)

|  | Model 1 (Unadjusted) | | | | | Model 2 (Adjusted) | | | | |
| --- | --- | --- | --- | --- | --- | --- | --- | --- | --- | --- |
| Variable | *B* | *SE* | *Exp(B)* | *Lower* | *Upper* | *B* | *SE* | *Exp(B)* | *Lower* | *Upper* |
| SSPI | 0.01 | 0.16 | 1.01 | 0.75 | 1.37 | -0.05 | 0.16 | 0.95 | 0.69 | 1.31 |
| PPM | -0.41 | 0.17 | 0.67 | 0.48 | 0.93 | -0.36 | 0.18 | 0.70 | 0.49 | 0.99 |
| UST | 0.03 | 0.17 | 1.03 | 0.74 | 1.44 | 0.07 | 0.18 | 1.08 | 0.76 | 1.52 |
| SCP | 0.43 | 0.19 | 1.53 | 1.06 | 2.21 | 0.48 | 0.20 | 1.61 | 1.10 | 2.37 |
| PCH | 0.13 | 0.18 | 1.14 | 0.80 | 1.62 | 0.09 | 0.19 | 1.10 | 0.76 | 1.58 |
| BSW | -0.26 | 0.19 | 0.77 | 0.53 | 1.12 | -0.30 | 0.20 | 0.74 | 0.50 | 1.10 |
| Sex |  |  |  |  |  | 0.46 | 0.52 | 1.58 | 0.57 | 4.43 |
| Age |  |  |  |  |  | -0.01 | 0.02 | 0.99 | 0.96 | 1.02 |
| Marital |  |  |  |  |  | -0.05 | 0.36 | 0.95 | 0.47 | 1.93 |
| Children |  |  |  |  |  | 0.06 | 0.36 | 1.06 | 0.52 | 2.17 |
| Job title |  |  |  |  |  | 0.67 | 0.32 | 1.95 | 1.05 | 3.63 |
| Work tempo |  |  |  |  |  | 0.24 | 0.27 | 1.27 | 0.75 | 2.15 |
| Staffing |  |  |  |  |  | 0.20 | 0.41 | 1.22 | 0.55 | 2.71 |

*Note: SSPI = Scheduling Support & Personal Influence; PPM = Prioritising Personal Motives; UST = Usability of Scheduling Tools; SCP = Scheduling as a Collective Process; PCH = Preference for Compressing Hours; BSW = Bad Schedule Warnings.*

** = p < .05; ** = p < .01; *** = p < .001, after Bonferroni correction*

Table S4. Summary of hierarchical logistic regression analysis for variables predicting long shifts (> 12 hours) (N = 238)

|  | Model 1 (Unadjusted) | | | | | Model 2 (Adjusted) | | | | |
| --- | --- | --- | --- | --- | --- | --- | --- | --- | --- | --- |
| Variable | *B* | *SE* | *Exp(B)* | *Lower* | *Upper* | *B* | *SE* | *Exp(B)* | *Lower* | *Upper* |
| SSPI | 0.01 | 0.17 | 1.01 | 0.73 | 1.40 | -0.08 | 0.18 | 0.92 | 0.65 | 1.30 |
| PPM | -0.09 | 0.18 | 0.91 | 0.65 | 1.29 | -0.09 | 0.19 | 0.91 | 0.63 | 1.33 |
| UST | 0.00 | 0.18 | 1.00 | 0.70 | 1.44 | -0.05 | 0.19 | 0.95 | 0.66 | 1.38 |
| SCP | 0.41 | 0.19 | 1.51 | 1.03 | 2.21 | 0.43 | 0.20 | 1.53 | 1.03 | 2.28 |
| PCH | 0.44 | 0.19 | 1.56 | 1.07 | 2.27 | 0.40 | 0.20 | 1.49 | 1.01 | 2.20 |
| BSW | -0.61 | 0.21 | **0.54*** | 0.36 | 0.82 | -0.63 | 0.22 | **0.53*** | 0.34 | 0.83 |
| Sex |  |  |  |  |  | 0.39 | 0.55 | 1.47 | 0.50 | 4.33 |
| Age |  |  |  |  |  | -0.02 | 0.02 | 0.98 | 0.95 | 1.01 |
| Marital |  |  |  |  |  | -0.79 | 0.38 | 0.46 | 0.22 | 0.96 |
| Children |  |  |  |  |  | -0.35 | 0.40 | 0.70 | 0.32 | 1.53 |
| Job title |  |  |  |  |  | -0.19 | 0.34 | 0.83 | 0.42 | 1.63 |
| Work tempo |  |  |  |  |  | -0.17 | 0.28 | 0.85 | 0.49 | 1.48 |
| Staffing |  |  |  |  |  | 0.54 | 0.47 | 1.72 | 0.69 | 4.29 |
| Part time |  |  |  |  |  | -0.11 | 0.62 | 0.90 | 0.27 | 3.05 |

*Note: SSPI = Scheduling Support & Personal Influence; PPM = Prioritising Personal Motives; UST = Usability of Scheduling Tools; SCP = Scheduling as a Collective Process; PCH = Preference for Compressing Hours; BSW = Bad Schedule Warnings.*

** = p < .05; ** = p < .01; *** = p < .001, after Bonferroni correction*

Table S5. Summary of hierarchical logistic regression analysis for variables predicting night shifts (N = 238)

|  | Model 1 (Unadjusted) | | | | | Model 2 (Adjusted) | | | | |
| --- | --- | --- | --- | --- | --- | --- | --- | --- | --- | --- |
| Variable | *B* | *SE* | *Exp(B)* | *Lower* | *Upper* | *B* | *SE* | *Exp(B)* | *Lower* | *Upper* |
| SSPI | 0.21 | 0.18 | 1.23 | 0.87 | 1.74 | 0.19 | 0.20 | 1.21 | 0.83 | 1.78 |
| PPM | 0.15 | 0.19 | 1.16 | 0.79 | 1.68 | 0.01 | 0.21 | 1.01 | 0.66 | 1.53 |
| UST | 0.13 | 0.19 | 1.14 | 0.78 | 1.66 | 0.03 | 0.21 | 1.03 | 0.68 | 1.57 |
| SCP | 0.37 | 0.23 | 1.45 | 0.93 | 2.26 | 0.38 | 0.24 | 1.46 | 0.91 | 2.35 |
| PCH | 0.31 | 0.21 | 1.37 | 0.91 | 2.06 | 0.32 | 0.22 | 1.37 | 0.88 | 2.13 |
| BSW | -0.24 | 0.22 | 0.79 | 0.51 | 1.20 | -0.17 | 0.24 | 0.85 | 0.53 | 1.35 |
| Sex |  |  |  |  |  | 1.56 | 1.07 | 4.77 | 0.58 | 39.13 |
| Age |  |  |  |  |  | -0.01 | 0.02 | 1.00 | 0.96 | 1.03 |
| Marital |  |  |  |  |  | -0.93 | 0.50 | 0.40 | 0.15 | 1.06 |
| Children |  |  |  |  |  | 0.48 | 0.46 | 1.62 | 0.66 | 3.96 |
| Job title |  |  |  |  |  | -1.06 | 0.37 | 0.35 | 0.17 | 0.72 |
| Work tempo |  |  |  |  |  | 0.35 | 0.32 | 1.41 | 0.75 | 2.66 |
| Staffing |  |  |  |  |  | 0.03 | 0.47 | 1.03 | 0.41 | 2.57 |
| Part time |  |  |  |  |  | -0.97 | 0.83 | 0.38 | 0.08 | 1.92 |

*Note: SSPI = Scheduling Support & Personal Influence; PPM = Prioritising Personal Motives; UST = Usability of Scheduling Tools; SCP = Scheduling as a Collective Process; PCH = Preference for Compressing Hours; BSW = Bad Schedule Warnings.*

** = p < .05; ** = p < .01; *** = p < .001, after Bonferroni correction*

Table S6. Summary of hierarchical logistic regression analysis for variables predicting quick returns (< 11 hours) (N = 238)

|  | Model 1 (Unadjusted) | | | | | Model 2 (Adjusted) | | | | |
| --- | --- | --- | --- | --- | --- | --- | --- | --- | --- | --- |
| Variable | *B* | *SE* | *Exp(B)* | *Lower* | *Upper* | *B* | *SE* | *Exp(B)* | *Lower* | *Upper* |
| SSPI | 0.24 | 0.28 | 1.27 | 0.73 | 2.21 | 0.55 | 0.34 | 1.74 | 0.89 | 3.40 |
| PPM | 0.02 | 0.30 | 1.02 | 0.57 | 1.85 | 0.27 | 0.38 | 1.31 | 0.62 | 2.76 |
| UST | -0.53 | 0.32 | 0.59 | 0.31 | 1.10 | -0.53 | 0.36 | 0.59 | 0.29 | 1.19 |
| SCP | -0.12 | 0.34 | 0.89 | 0.46 | 1.71 | -0.37 | 0.39 | 0.69 | 0.32 | 1.49 |
| PCH | 0.22 | 0.31 | 1.25 | 0.67 | 2.30 | 0.46 | 0.37 | 1.59 | 0.77 | 3.29 |
| BSW | 0.42 | 0.36 | 1.52 | 0.75 | 3.07 | 0.07 | 0.41 | 1.08 | 0.48 | 2.41 |
| Sex |  |  |  |  |  | -0.31 | 1.18 | 0.74 | 0.07 | 7.42 |
| Age |  |  |  |  |  | 0.13 | 0.04 | 1.14 | 1.06 | 1.23 |
| Marital |  |  |  |  |  | 0.44 | 0.77 | 1.55 | 0.34 | 7.08 |
| Children |  |  |  |  |  | 0.07 | 0.81 | 1.07 | 0.22 | 5.19 |
| Job title |  |  |  |  |  | -0.26 | 0.62 | 0.77 | 0.23 | 2.61 |
| Work tempo |  |  |  |  |  | -0.60 | 0.58 | 0.55 | 0.18 | 1.72 |
| Staffing |  |  |  |  |  | 0.12 | 0.91 | 1.13 | 0.19 | 6.74 |
| Part time |  |  |  |  |  | 19.80 | 8933.65 | 395397721.18 | 0.00 | . |

*Note: SSPI = Scheduling Support & Personal Influence; PPM = Prioritising Personal Motives; UST = Usability of Scheduling Tools; SCP = Scheduling as a Collective Process; PCH = Preference for Compressing Hours; BSW = Bad Schedule Warnings.*

** = p < .05; ** = p < .01; *** = p < .001, after Bonferroni correction*

Table S7. Summary of hierarchical logistic regression analysis for variables predicting single days off (N = 238)

|  | Model 1 (Unadjusted) | | | | | Model 2 (Adjusted) | | | | |
| --- | --- | --- | --- | --- | --- | --- | --- | --- | --- | --- |
| Variable | *B* | *SE* | *Exp(B)* | *Lower* | *Upper* | *B* | *SE* | *Exp(B)* | *Lower* | *Upper* |
| SSPI | -0.12 | 0.18 | 0.89 | 0.63 | 1.26 | -0.05 | 0.19 | 0.95 | 0.66 | 1.37 |
| PPM | -0.08 | 0.19 | 0.92 | 0.64 | 1.33 | -0.12 | 0.20 | 0.89 | 0.60 | 1.31 |
| UST | 0.30 | 0.19 | 1.35 | 0.93 | 1.97 | 0.23 | 0.20 | 1.26 | 0.85 | 1.85 |
| SCP | 0.17 | 0.21 | 1.19 | 0.79 | 1.79 | 0.16 | 0.22 | 1.17 | 0.77 | 1.79 |
| PCH | -0.17 | 0.21 | 0.85 | 0.57 | 1.27 | -0.08 | 0.21 | 0.92 | 0.61 | 1.40 |
| BSW | -0.40 | 0.22 | 0.67 | 0.44 | 1.03 | -0.39 | 0.23 | 0.68 | 0.43 | 1.07 |
| Sex |  |  |  |  |  | 0.16 | 0.58 | 1.17 | 0.38 | 3.63 |
| Age |  |  |  |  |  | 0.00 | 0.02 | 1.00 | 0.97 | 1.04 |
| Marital |  |  |  |  |  | -0.57 | 0.39 | 0.57 | 0.27 | 1.22 |
| Children |  |  |  |  |  | -0.25 | 0.42 | 0.78 | 0.34 | 1.76 |
| Job title |  |  |  |  |  | -0.49 | 0.36 | 0.61 | 0.30 | 1.25 |
| Work tempo |  |  |  |  |  | 0.06 | 0.30 | 1.06 | 0.59 | 1.90 |
| Staffing |  |  |  |  |  | -0.48 | 0.42 | 0.62 | 0.27 | 1.40 |
| Part time |  |  |  |  |  | 1.42 | 1.08 | 4.15 | 0.50 | 34.43 |

*Note: SSPI = Scheduling Support & Personal Influence; PPM = Prioritising Personal Motives; UST = Usability of Scheduling Tools; SCP = Scheduling as a Collective Process; PCH = Preference for Compressing Hours; BSW = Bad Schedule Warnings.*

** = p < .05; ** = p < .01; *** = p < .001, after Bonferroni correction*

Table S8. Summary of hierarchical logistic regression analysis for variables predicting > 4 consecutive shifts (N = 238)

|  | Model 1 (Unadjusted) | | | | | Model 2 (Adjusted) | | | | |
| --- | --- | --- | --- | --- | --- | --- | --- | --- | --- | --- |
| Variable | *B* | *SE* | *Exp(B)* | *Lower* | *Upper* | *B* | *SE* | *Exp(B)* | *Lower* | *Upper* |
| SSPI | -0.04 | 0.23 | 0.97 | 0.61 | 1.52 | -0.10 | 0.25 | 0.91 | 0.55 | 1.49 |
| PPM | 0.04 | 0.24 | 1.04 | 0.65 | 1.67 | -0.08 | 0.25 | 0.92 | 0.56 | 1.51 |
| UST | 0.13 | 0.25 | 1.14 | 0.70 | 1.85 | 0.16 | 0.26 | 1.18 | 0.71 | 1.95 |
| SCP | 0.54 | 0.26 | 1.71 | 1.02 | 2.86 | 0.57 | 0.29 | 1.77 | 1.00 | 3.14 |
| PCH | 0.03 | 0.26 | 1.03 | 0.62 | 1.72 | 0.09 | 0.28 | 1.09 | 0.63 | 1.87 |
| BSW | -0.04 | 0.27 | 0.96 | 0.56 | 1.63 | -0.10 | 0.29 | 0.90 | 0.51 | 1.61 |
| Sex |  |  |  |  |  | 0.76 | 0.66 | 2.14 | 0.59 | 7.78 |
| Age |  |  |  |  |  | 0.00 | 0.02 | 1.00 | 0.96 | 1.05 |
| Marital |  |  |  |  |  | 1.61 | 0.80 | 4.98 | 1.04 | 23.90 |
| Children |  |  |  |  |  | 0.53 | 0.53 | 1.70 | 0.60 | 4.82 |
| Job title |  |  |  |  |  | 0.10 | 0.48 | 1.10 | 0.43 | 2.82 |
| Work tempo |  |  |  |  |  | -0.19 | 0.39 | 0.83 | 0.39 | 1.77 |
| Staffing |  |  |  |  |  | 0.52 | 0.69 | 1.69 | 0.43 | 6.54 |
| Part time |  |  |  |  |  | 19.41 | 9816.94 | 269779279.24 | 0.00 | . |

*Note: SSPI = Scheduling Support & Personal Influence; PPM = Prioritising Personal Motives; UST = Usability of Scheduling Tools; SCP = Scheduling as a Collective Process; PCH = Preference for Compressing Hours; BSW = Bad Schedule Warnings.*

** = p < .05; ** = p < .01; *** = p < .001, after Bonferroni correction*

Table S9. Summary of hierarchical logistic regression analysis for variables predicting < 28 hours recovery after last night shift (N = 238)

|  | Model 1 (Unadjusted) | | | | | Model 2 (Adjusted) | | | | |
| --- | --- | --- | --- | --- | --- | --- | --- | --- | --- | --- |
| Variable | *B* | *SE* | *Exp(B)* | *Lower* | *Upper* | *B* | *SE* | *Exp(B)* | *Lower* | *Upper* |
| SSPI | -0.33 | 0.16 | 0.72 | 0.53 | 0.98 | -0.34 | 0.17 | 0.71 | 0.51 | 0.99 |
| PPM | -0.13 | 0.17 | 0.88 | 0.63 | 1.22 | -0.18 | 0.18 | 0.84 | 0.58 | 1.19 |
| UST | 0.07 | 0.17 | 1.07 | 0.77 | 1.50 | 0.07 | 0.18 | 1.07 | 0.76 | 1.52 |
| SCP | 0.12 | 0.19 | 1.13 | 0.78 | 1.63 | 0.06 | 0.20 | 1.06 | 0.72 | 1.57 |
| PCH | -0.17 | 0.18 | 0.84 | 0.59 | 1.20 | -0.14 | 0.19 | 0.87 | 0.60 | 1.26 |
| BSW | -0.01 | 0.19 | 0.99 | 0.68 | 1.43 | -0.09 | 0.20 | 0.92 | 0.62 | 1.36 |
| Sex |  |  |  |  |  | 0.78 | 0.51 | 2.17 | 0.81 | 5.86 |
| Age |  |  |  |  |  | 0.01 | 0.02 | 1.01 | 0.98 | 1.04 |
| Marital |  |  |  |  |  | 0.80 | 0.42 | 2.22 | 0.99 | 5.01 |
| Children |  |  |  |  |  | -0.25 | 0.38 | 0.78 | 0.37 | 1.64 |
| Job title |  |  |  |  |  | -0.08 | 0.32 | 0.92 | 0.49 | 1.73 |
| Work tempo |  |  |  |  |  | -0.17 | 0.27 | 0.84 | 0.49 | 1.43 |
| Staffing |  |  |  |  |  | 0.30 | 0.41 | 1.36 | 0.60 | 3.05 |
| Part time |  |  |  |  |  | 1.26 | 0.80 | 3.54 | 0.74 | 16.89 |

*Note: SSPI = Scheduling Support & Personal Influence; PPM = Prioritising Personal Motives; UST = Usability of Scheduling Tools; SCP = Scheduling as a Collective Process; PCH = Preference for Compressing Hours; BSW = Bad Schedule Warnings.*

** = p < .05; ** = p < .01; *** = p < .001, after Bonferroni correction*

Table S10. Summary of hierarchical logistic regression analysis for variables predicting ≥ 4 consecutive night shifts (N = 238)

|  | Model 1 (Unadjusted) | | | | | Model 2 (Adjusted) | | | | |
| --- | --- | --- | --- | --- | --- | --- | --- | --- | --- | --- |
| Variable | *B* | *SE* | *Exp(B)* | *Lower* | *Upper* | *B* | *SE* | *Exp(B)* | *Lower* | *Upper* |
| SSPI | 0.34 | 0.20 | 1.41 | 0.95 | 2.08 | 0.29 | 0.21 | 1.33 | 0.88 | 2.02 |
| PPM | -0.34 | 0.21 | 0.71 | 0.47 | 1.08 | -0.37 | 0.23 | 0.69 | 0.44 | 1.08 |
| UST | 0.18 | 0.22 | 1.20 | 0.79 | 1.83 | 0.17 | 0.22 | 1.19 | 0.77 | 1.83 |
| SCP | 0.20 | 0.22 | 1.23 | 0.79 | 1.89 | 0.24 | 0.23 | 1.27 | 0.81 | 1.99 |
| PCH | 0.39 | 0.23 | 1.48 | 0.94 | 2.34 | 0.41 | 0.24 | 1.51 | 0.94 | 2.42 |
| BSW | -0.36 | 0.24 | 0.70 | 0.43 | 1.12 | -0.38 | 0.26 | 0.69 | 0.42 | 1.13 |
| Sex |  |  |  |  |  | 0.40 | 0.65 | 1.49 | 0.42 | 5.28 |
| Age |  |  |  |  |  | -0.01 | 0.02 | 0.99 | 0.95 | 1.03 |
| Marital |  |  |  |  |  | -0.43 | 0.42 | 0.65 | 0.28 | 1.50 |
| Children |  |  |  |  |  | 0.48 | 0.44 | 1.61 | 0.69 | 3.78 |
| Job title |  |  |  |  |  | 0.15 | 0.41 | 1.16 | 0.53 | 2.57 |
| Work tempo |  |  |  |  |  | 0.39 | 0.33 | 1.48 | 0.77 | 2.82 |
| Staffing |  |  |  |  |  | 0.06 | 0.53 | 1.06 | 0.38 | 2.98 |
| Part time |  |  |  |  |  | 0.36 | 0.82 | 1.43 | 0.29 | 7.11 |

*Note: SSPI = Scheduling Support & Personal Influence; PPM = Prioritising Personal Motives; UST = Usability of Scheduling Tools; SCP = Scheduling as a Collective Process; PCH = Preference for Compressing Hours; BSW = Bad Schedule Warnings.*

** = p < .05; ** = p < .01; *** = p < .001, after Bonferroni correction*

Table S11. Summary of hierarchical logistic regression analysis for variables predicting satisfaction with work hours (N = 248)

|  | Model 1 (Unadjusted) | | | | | Model 2 (Adjusted) | | | | |
| --- | --- | --- | --- | --- | --- | --- | --- | --- | --- | --- |
| Variable | *B* | *SE* | *Exp(B)* | *Lower* | *Upper* | *B* | *SE* | *Exp(B)* | *Lower* | *Upper* |
| SSPI | 0.66 | 0.19 | **1.93**** | 1.34 | 2.78 | 0.65 | 0.20 | **1.92**** | 1.30 | 2.82 |
| PPM | 0.01 | 0.18 | 1.01 | 0.71 | 1.44 | -0.11 | 0.20 | 0.90 | 0.61 | 1.32 |
| UST | 0.18 | 0.19 | 1.20 | 0.82 | 1.74 | 0.14 | 0.20 | 1.15 | 0.78 | 1.70 |
| SCP | -0.09 | 0.21 | 0.92 | 0.61 | 1.38 | -0.11 | 0.22 | 0.90 | 0.59 | 1.38 |
| PCH | 0.05 | 0.20 | 1.05 | 0.70 | 1.57 | 0.02 | 0.21 | 1.02 | 0.67 | 1.54 |
| BSW | 0.09 | 0.22 | 1.09 | 0.71 | 1.68 | 0.18 | 0.24 | 1.20 | 0.76 | 1.90 |
| Sex |  |  |  |  |  | -0.20 | 0.58 | 0.82 | 0.26 | 2.56 |
| Age |  |  |  |  |  | -0.03 | 0.02 | 0.97 | 0.94 | 1.00 |
| Marital |  |  |  |  |  | 0.29 | 0.43 | 1.33 | 0.57 | 3.09 |
| Children |  |  |  |  |  | -0.53 | 0.43 | 0.59 | 0.26 | 1.36 |
| Job title |  |  |  |  |  | -0.27 | 0.37 | 0.77 | 0.37 | 1.57 |
| Work tempo |  |  |  |  |  | 0.03 | 0.31 | 1.03 | 0.57 | 1.88 |
| Staffing |  |  |  |  |  | -0.08 | 0.47 | 0.92 | 0.37 | 2.33 |
| Part time |  |  |  |  |  | 0.06 | 0.69 | 1.07 | 0.28 | 4.12 |

*Note: SSPI = Scheduling Support & Personal Influence; PPM = Prioritising Personal Motives; UST = Usability of Scheduling Tools; SCP = Scheduling as a Collective Process; PCH = Preference for Compressing Hours; BSW = Bad Schedule Warnings.*

** = p < .05; ** = p < .01; *** = p < .001, after Bonferroni correction*

Table S12. Summary of hierarchical logistic regression analysis for variables predicting enjoying the job (N = 248)

|  | Model 1 (Unadjusted) | | | | | Model 2 (Adjusted) | | | | |
| --- | --- | --- | --- | --- | --- | --- | --- | --- | --- | --- |
| Variable | *B* | *SE* | *Exp(B)* | *Lower* | *Upper* | *B* | *SE* | *Exp(B)* | *Lower* | *Upper* |
| SSPI | 0.54 | 0.42 | 1.71 | 0.73 | 3.99 | 0.80 | 0.57 | 2.22 | 0.68 | 7.24 |
| PPM | 0.01 | 0.40 | 1.01 | 0.46 | 2.24 | -0.06 | 0.47 | 0.94 | 0.37 | 2.42 |
| UST | -0.37 | 0.42 | 0.69 | 0.30 | 1.59 | -0.42 | 0.46 | 0.66 | 0.26 | 1.62 |
| SCP | 0.26 | 0.50 | 1.29 | 0.46 | 3.61 | 0.30 | 0.48 | 1.35 | 0.51 | 3.54 |
| PCH | 0.00 | 0.43 | 1.00 | 0.43 | 2.32 | 0.06 | 0.45 | 1.06 | 0.44 | 2.57 |
| BSW | 0.22 | 0.52 | 1.25 | 0.45 | 3.49 | 0.37 | 0.56 | 1.44 | 0.47 | 4.40 |
| Sex |  |  |  |  |  | -18.51 | 8383.61 | 0.00 | 0.00 | . |
| Age |  |  |  |  |  | 0.01 | 0.04 | 1.01 | 0.94 | 1.08 |
| Marital |  |  |  |  |  | 0.23 | 0.97 | 1.26 | 0.18 | 8.64 |
| Children |  |  |  |  |  | 0.58 | 0.81 | 1.78 | 0.36 | 8.73 |
| Job title |  |  |  |  |  | -0.66 | 0.84 | 0.52 | 0.10 | 2.74 |
| Work tempo |  |  |  |  |  | -0.03 | 0.64 | 0.97 | 0.27 | 3.44 |
| Staffing |  |  |  |  |  | -1.15 | 1.05 | 0.32 | 0.04 | 2.68 |
| Part time |  |  |  |  |  | -0.32 | 1.23 | 0.73 | 0.07 | 8.13 |

*Note: SSPI = Scheduling Support & Personal Influence; PPM = Prioritising Personal Motives; UST = Usability of Scheduling Tools; SCP = Scheduling as a Collective Process; PCH = Preference for Compressing Hours; BSW = Bad Schedule Warnings.*

** = p < .05; ** = p < .01; *** = p < .001, after Bonferroni correction*

Table S13. Summary of hierarchical logistic regression analysis for variables predicting satisfaction with influence over work hours (N = 248)

|  | Model 1 (Unadjusted) | | | | | Model 2 (Adjusted) | | | | |
| --- | --- | --- | --- | --- | --- | --- | --- | --- | --- | --- |
| Variable | *B* | *SE* | *Exp(B)* | *Lower* | *Upper* | *B* | *SE* | *Exp(B)* | *Lower* | *Upper* |
| SSPI | 0.81 | 0.22 | **2.25***** | 1.46 | 3.49 | 0.71 | 0.23 | **2.04**** | 1.29 | 3.22 |
| PPM | 0.38 | 0.21 | 1.46 | 0.97 | 2.19 | 0.38 | 0.22 | 1.47 | 0.95 | 2.28 |
| UST | 0.21 | 0.22 | 1.23 | 0.81 | 1.89 | 0.17 | 0.22 | 1.18 | 0.76 | 1.83 |
| SCP | 0.13 | 0.23 | 1.14 | 0.73 | 1.78 | 0.07 | 0.24 | 1.07 | 0.67 | 1.73 |
| PCH | 0.31 | 0.23 | 1.37 | 0.88 | 2.14 | 0.34 | 0.24 | 1.41 | 0.89 | 2.25 |
| BSW | 0.39 | 0.25 | 1.48 | 0.90 | 2.44 | 0.36 | 0.27 | 1.44 | 0.85 | 2.43 |
| Sex |  |  |  |  |  | -0.28 | 0.65 | 0.75 | 0.21 | 2.69 |
| Age |  |  |  |  |  | -0.02 | 0.02 | 0.98 | 0.94 | 1.02 |
| Marital |  |  |  |  |  | -0.03 | 0.48 | 0.97 | 0.38 | 2.47 |
| Children |  |  |  |  |  | -0.43 | 0.48 | 0.65 | 0.25 | 1.69 |
| Job title |  |  |  |  |  | 0.05 | 0.41 | 1.06 | 0.47 | 2.36 |
| Work tempo |  |  |  |  |  | -0.36 | 0.36 | 0.70 | 0.34 | 1.43 |
| Staffing |  |  |  |  |  | 1.04 | 0.79 | 2.84 | 0.61 | 13.28 |
| Part time |  |  |  |  |  | 15.91 | 8587.13 | 8103938.98 | 0.00 | . |

*Note: SSPI = Scheduling Support & Personal Influence; PPM = Prioritising Personal Motives; UST = Usability of Scheduling Tools; SCP = Scheduling as a Collective Process; PCH = Preference for Compressing Hours; BSW = Bad Schedule Warnings.*

** = p < .05; ** = p < .01; *** = p < .001, after Bonferroni correction*

Table S14. Summary of hierarchical logistic regression analysis for variables predicting satisfaction with thoughts of leaving the workplace (N = 248)

|  | Model 1 (Unadjusted) | | | | | Model 2 (Adjusted) | | | | |
| --- | --- | --- | --- | --- | --- | --- | --- | --- | --- | --- |
| Variable | *B* | *SE* | *Exp(B)* | *Lower* | *Upper* | *B* | *SE* | *Exp(B)* | *Lower* | *Upper* |
| SSPI | 0.72 | 0.17 | **2.06**** | 1.47 | 2.90 | 0.81 | 0.19 | **2.24**** | 1.54 | 3.28 |
| PPM | 0.05 | 0.17 | 1.05 | 0.75 | 1.46 | -0.01 | 0.19 | 0.99 | 0.69 | 1.43 |
| UST | -0.07 | 0.18 | 0.93 | 0.66 | 1.32 | -0.16 | 0.19 | 0.85 | 0.58 | 1.23 |
| SCP | -0.15 | 0.20 | 0.86 | 0.58 | 1.27 | -0.16 | 0.21 | 0.85 | 0.56 | 1.29 |
| PCH | -0.24 | 0.19 | 0.79 | 0.54 | 1.15 | -0.22 | 0.20 | 0.80 | 0.54 | 1.19 |
| BSW | 0.14 | 0.21 | 1.14 | 0.76 | 1.72 | 0.17 | 0.22 | 1.18 | 0.76 | 1.82 |
| Sex |  |  |  |  |  | -0.33 | 0.61 | 0.72 | 0.22 | 2.37 |
| Age |  |  |  |  |  | 0.00 | 0.02 | 1.00 | 0.97 | 1.04 |
| Marital |  |  |  |  |  | -0.71 | 0.38 | 0.49 | 0.23 | 1.04 |
| Children |  |  |  |  |  | 0.58 | 0.38 | 1.78 | 0.85 | 3.74 |
| Job title |  |  |  |  |  | -0.68 | 0.36 | 0.51 | 0.25 | 1.02 |
| Work tempo |  |  |  |  |  | -0.17 | 0.28 | 0.85 | 0.49 | 1.48 |
| Staffing |  |  |  |  |  | -0.46 | 0.44 | 0.64 | 0.27 | 1.50 |
| Part time |  |  |  |  |  | 0.50 | 0.72 | 1.65 | 0.40 | 6.73 |

*Note: SSPI = Scheduling Support & Personal Influence; PPM = Prioritising Personal Motives; UST = Usability of Scheduling Tools; SCP = Scheduling as a Collective Process; PCH = Preference for Compressing Hours; BSW = Bad Schedule Warnings.*

** = p < .05; ** = p < .01; *** = p < .001, after Bonferroni correction*
